# Whey Protein Powder Analysis by Mid-Infrared Spectroscopy

**DOI:** 10.3390/foods10051033

**Published:** 2021-05-10

**Authors:** Rose Saxton, Owen M. McDougal

**Affiliations:** 1 Biomolecular Sciences Graduate Program, Boise State University, Boise, ID 83725, USA; rosesaxton@u.boisestate.edu; 2 Department of Chemistry and Biochemistry, Boise State University, Boise, ID 83725, USA

**Keywords:** milk and dairy products, whey protein, spectroscopic analysis, mid-infrared spectroscopy, adulteration, Kjeldahl

## Abstract

There is an ever-expanding number of high protein dietary supplements marketed as beneficial to athletes, body builders, infant formulas, elder care, and animal feed. Consumers will pay more for products with high protein per serving data on their nutritional labels, making the accurate reporting of protein content critical to customer confidence. The Kjeldahl method (KM) is the industry standard to quantitate dairy proteins, but the result is based on nitrogen content, which is an approximation of nitrogen attributable to protein in milk. Product tampering by third-party manufacturers is an issue, due to the lack of United States Food and Drug Administration regulation of nutraceutical products, permitting formulators to add low-cost nitrogen-containing components to artificially inflate the KM approximated protein content in products. Optical spectroscopy is commonly used for quality control measurements and has been identified as having the potential to complement the KM as a more nuanced testing measure of dairy protein. Mid-infrared (MIR) spectroscopy spectra of eight protein standards provided qualitative characterization of each protein by amide I and amide II peak absorbance wavenumber. Protein doping experiments revealed that as protein amounts were increased, the amide I/II peak shape changed from the broad protein powder peaks to the narrower peaks characteristic of the individual protein. Amino acid doping experiments with lysine, glutamic acid, and glycine, determined the limit of detection by MIR spectroscopy as 25%, suggesting that MIR spectroscopy can provide product quality assurance complementary to dairy protein measurement by the KM.

## 1. Introduction

Dietary protein is required to provide amino acid building blocks critical to growth and development, catalysts for biochemical reactions and mechanical support of tissues [1]. The two most abundant dairy proteins are casein and whey. Casein is the primary constituent of milk, used to make cheese, while whey is most often made into yogurt or spray-dried for use in nutritional supplements, among other products. Whey is a homogenous mixture of many proteins, comprised of 63–50% β-lactoglobulin, 20% α-lactalbumin, 8–6% bovine serum albumin, and 1% immunoglobulin G [2,3,4,5,6,7,8,9,10]. Each whey protein contains all of the essential amino acids, which makes them valuable ingredients in protein powder formulations, but not all protein sources are created equal, with milk, eggs, and legumes emerging as natural sources of premium protein for dietary supplements. A problem that threatens protein suppliers is the introduction of low value amino acids or inexpensive proteins that are used to inflate reported protein content in formulated products. Proteins, particularly dairy proteins, are a complete source of all amino acids in the right proportion to provide optimal nutritional benefit to the consumer.

When single amino acids are introduced into a nutritional product, they are generally inexpensive, non-essential amino acids like glycine or glutamic acid, that the human body naturally produces in sufficient quantity such that additional ingestion provides no perceived benefit. The use of a less expensive amino acid, like glycine, to increase the nitrogen content of protein products is a practice that has made the news in recent years. In 2014, a class action lawsuit was filed against the makers of “Body Fortress Super Advanced Whey Protein,” claiming that free amino acids including glutamine, creatine, glycine, and taurine were used to increase the purported protein content of the product [11]. The ingredient label stated that each serving contained 30 g of protein, but chemical analysis of the product identified only 21.5 g of whey protein per serving, a 28.3% difference between reported protein and actual protein. Because not all countries have stringent food regulatory agencies, the practice of adulterating milk products with non-protein, nitrogen-containing compounds, is commonly encountered. In 2008, Chinese milk powder was found to contain melamine, which led to six infant deaths [12]. The World Health Organization (WHO) reports that melamine and cyanuric acid alone, in low doses, are not harmful, however the combination of the two, which produces melamine cyanurate, is toxic [13]. Melamine cyanurate is unable to be metabolized, leading to crystal formation in the kidneys that are not able to be eliminated, eventually resulting in death if not addressed. Melamine has no dietary benefit, and in the case of the contaminated Chinese milk powder, was used solely to increase the nitrogen content of the milk powder; one molecule of melamine contains six nitrogen atoms.

The traditional approach to measure protein content in food is the Kjeldahl method (KM), which has been updated over the years to better distinguish between non-casein-nitrogen (NCN) and non-protein-nitrogen (NPN) in milk [14,15]. For whole milk, AOAC method **991.20**, which uses TCA to precipitate protein, is generally followed to determine total nitrogen content and NCN. Additional AOAC methods have been developed for the analysis of milk and milk powders to measure total protein, NPN, and NCN, using acetic acid/sodium acetate to accomplish protein precipitation [16,17,18,19]. The precipitation of albumin and globulin proteins can also be accomplished with TCA by lowering the pH of milk to 4.7 [20]. Both the traditional and modified KM protocol take hours to complete and use predetermined dairy conversion factors to calculate protein content for milk and milk products.

Mid-infrared (MIR) spectroscopy has been used for the evaluation of fat, protein, and casein content in cow’s milk, and to monitor product storage tolerance by measuring adducts formed by lipolysis and proteolysis over time [21,22]. Additional scientific studies of whey proteins have included proteomic and sodium dodecyl sulphate–polyacrylamide gel electrophoresis (SDS-PAGE) analysis, as well as quantification by reversed phase-high pressure liquid chromatography (HPLC), high-pressure liquid chromatography-electrospray ionization-quadrupole time-of-flight mass spectrometry (LC-ESI-Q-TOF MS), and confocal Raman microscopy [23,24,25,26,27]. While MIR spectroscopy requires minimal sample preparation time, relatively inexpensive instrumentation, and offers rapid analysis, the proteomic and liquid chromatography studies are appreciably more resource intensive.

There exists a need in the food industry for rapid testing techniques that address quality assurance and quality control measures for dietary supplements. To demonstrate the potential for MIR spectroscopy to be used for quality assurance testing of whey protein dietary supplements, a series of amino acids, proteins, and protein powder (whey-based and plant-based) product samples were tested by KM and MIR. The KM was used for the analysis of the amino acid lysine, and five commercially available protein powders, including four whey-based and one plant-based product; according to the three-step protocol: digestion, distillation, and titration [28,29]. Three different quantities (0.5, 1.0, and 2.0 g) were used to evaluate how increasing sample size would affect the measurement of nitrogen and calculation of protein content. MIR was used to further investigate the individual proteins in the protein powders tested in this study, where four whey (β-lactoglobulin, α-lactalbumin, bovine serum albumin (BSA), and immunoglobulin G (IgG)), casein, egg, and two plant-proteins (brown rice and pea) were evaluated. MIR spectra were obtained for the five protein powders, focusing on four spectral regions (amide I, amide II, carbohydrate, and lipid). KM and MIR analysis were also used to evaluate protein doping of a whey protein powder with increasing amounts of BSA. Lastly, MIR was used to evaluate amino acid doping of a whey protein powder, where one whey protein powder was spiked with known amounts of three different amino acids (glutamic acid, lysine, and glycine).

## 2. Infrared (IR) Spectroscopy

**Figure 1:**
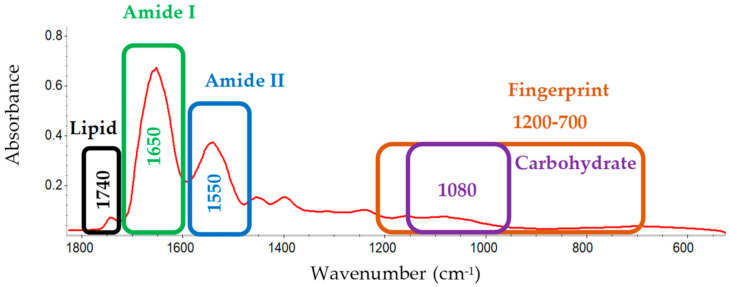
Full pea protein mid-infrared spectrum, with wavenumber identifiers. Mid-IR spectrum highlighting regions discussed: lipid region (1740 cm^−1^) (black), amide I region (1700–1600 cm^−1^) (green), amide II region (1580–1510 cm^−1^) (blue), fingerprint region (1200–700 cm^−1^) (orange), and carbohydrate region (1080 cm^−1^) (purple).

Infrared spectroscopy offers rapid analysis of amide bond absorbance at precise wavenumbers for amino acids in proteins and can differentiate proteins by monitoring signature absorbance frequencies. MIR spectroscopy passes light at wavelengths ranging from 2.5–25 µm (wavenumbers: 4000–400 cm^−1^, respectively) through a sample, permitting detection of absorbance frequency based on characteristic bond vibration. When infrared light hits the bonds of a protein, wavelengths of light are absorbed characteristic to the vibrational frequency of the bonded atoms and a signal is produced. The absorbance signature of one protein can be differentiated from other proteins dependent on the unique absorbance pattern for each. When distinguishing proteins by MIR, the amide I (1700–1600 cm^−1^) and amide II (1580–1510 cm^−1^) regions of the spectrum have been identified as the most useful [30]. Gallagher describes the amide I band as being due to the carbonyl stretching vibration between 1700–1600 cm^−1^, and the amide II band is due to the N-H bending vibration between 1580–1510 cm^−1^. Krimm and Bandekar describe the amide I/II bands in greater detail; with the amide I band being mainly due to the C-O stretching vibration but also the C-N stretching and C-C-N deformation vibrations, and the amide II being a combination of the N-H in-plane bending, C-N stretching, C-O in-plane bending, C-C stretching, and N-C stretching vibrations [31]. Amide I and II band intensity are also, in-part, due to the amino acid side-chain groups which are influenced by pH [32]. The MIR literature associated with protein product analysis suggests proteins may also be distinguished from one other using two other product components; including lipids (≈1743 cm^−1^) and carbohydrates (≈1080 cm^−1^) [33]. The lipid peak at 1743 cm^−1^ is characteristic of the ester C = O stretching from triglycerides [34]. The carbohydrate peak at 1080 cm^−1^ is due to the C-O stretch common to all polyhydroxy aldehydes and ketones [35]. The fingerprint region (1200–700 cm^−1^) is used for structural confirmation [36]. The five regions discussed (amide I, amide II, lipid, carbohydrate, and fingerprint) are shown in Figure 1.

The make-up of a protein starts with the individual amino acids that come together and are joined by the amino group of one binding with the carboxyl group of another, through a condensation reaction, forming a peptide bond; also known as an amide bond [37]. The amino acid sequence of a protein forms the primary protein structure and contributes to the three-dimensional structure for that protein. The unique sequence/structure attribute for a given protein can be measured as a characteristic IR “fingerprint”, distinct from other proteins by the pattern of maximum absorbance wavenumbers and absorbance signal magnitudes in the IR spectrum. For example, both α-lactalbumin and BSA are dominated by α-helical secondary arrangement, but their size and structural differences are sufficiently diverse to provide amide I absorbance bands of unique wavenumber for them to be distinguished from one another by MIR spectroscopy [38]. Quality assurance for a whey protein dietary supplement that has been doped with amino acids can be readily achieved using MIR spectroscopy, because the amino acids do not have peptide bonds of unique signature absorbance, whereas the whey proteins do. Although individual amino acids absorb light within the amide I and amide II regions of the spectrum, their absorbance is less distinctive and more variable in pattern, making them easily discerned from proteins [39].

## 3. Materials and Methods

### 3.1. Equipment

Protein powder weights were taken on a Torbal AGZN200 top loading balance to the nearest 0.0001 g. Infrared (IR) spectra were recorded using a Nicolet^TM^ iS20 FT-IR spectrometer equipped with a Nicolet^TM^ iZ10 module and OMNIC 9 Software Suite. The IR spectrometer was used in conjunction with an attenuated total reflectance (ATR) diamond plate that was cleaned with isopropanol, allowed to dry, and a background spectrum recorded prior to sample runs. In each case, the background spectrum was subtracted from the protein powder spectrum, to generate a true sample spectrum. Protein powder samples were loaded on the surface of the ATR accessory and a force probe was tightened to ensure adequate contact with the crystal; a total of three spectra were collected for each protein powder, after every sample analysis, the crystal was cleaned with isopropanol and a new sample was analyzed. Collection parameters included 512 scans at a resolution of 4 cm^−1^, with data spacing at 0.482 cm^−1^, using a DTGS KBr detector and KBr beam splitter. Spectra were collected using Blackman–Harris apodization and Mertz phase correction. After data collection, the advanced ATR-correction and auto optimization features of Thermo Scientific™ OMNIC™ software were applied to all spectra. The Blackman–Harris apodization increases the signal-to-noise ratio and the Mertz phase correction ensures that a true sample spectrum is generated. The advanced ATR-correction feature includes correcting for variations in the depth of penetration and absorption band shifts between samples and the auto optimization feature includes baseline correction, blanking the saturated peaks, and smoothing and normalizing each spectrum. To generate graphs for data, CVS files were downloaded from OMNIC^TM^, consolidated into one file, and saved in Excel format. The Excel files were imported into RStudio where figures were constructed [40].

### 3.2. Materials, Samples and Standards

Commercially available whey protein powders were purchased from BodyBuilding.com and the pea protein powder was purchased from a local grocery store. The protein standards β-lactoglobulin (≥90%, Catalog #L3908-5G), α-lactalbumin (≥85%, Catalog #50-176-5110), and IgG (≥95%, Catalog #I5506-10MG) were purchased from Sigma Aldrich. The protein standard BSA was purchased from Fisher Scientific (Catalog #BP9700100). The pea (80%) and brown rice (80%) proteins were purchased from Amazon.com and both were sourced from Terrasoul Superfoods. The amino acid glycine at 99% purity was purchased from Leco.com (Part #502-211). The L-lysine monohydrochloride (98.5–100.5%, Catalog #BP386-100), L-glutamic acid (≥99%, Catalog #A125-100), and skim milk powder (Catalog #OXLP0031B) were purchased from Fisher Scientific. All chemicals were purchased from Fisher Scientific, including sodium hydroxide pellets (Catalog #S318-500), boric acid powder (Product #A74-1), hydrochloric acid (Catalog #A144S-500), and ammonium sulfate (99.999%, Catalog #AA1063909).

### 3.3. Reagents for the Kjeldahl Method

Unless otherwise stated, all reagents were purchased from Fisher Scientific. The reagents used for the Kjeldahl method included concentrated sulfuric acid (95–98%, Product #A484-212), and Kjeldahl catalyst tablets (FisherTab^TM^ CT-37 Kjeldahl Tablets, Product #K3011000); each tablet has a mass of 3.9 g and consists of 3.5 g K_2_SO_4_ and 0.4 g CuSO_4_. The protein digestion mixture used for Kjeldahl experiments was prepared by combining two FisherTabs^TM^, 5, 10, or 15 mL of sulfuric acid, and a 0.5, 1.0, or 2.0 g sample of protein powder. After digestion, deionized (DI) water was added to dilute the mixture to prevent precipitation. Solutions (weight/volume) of 40% sodium hydroxide, 4% boric acid, 0.1 M sodium hydroxide, and 0.1 M hydrochloric acid were prepared. To 1.0 L of 4% boric acid receiving solution, was added 1.5–2.0 mL of a bromocresol green-methyl red mixed indicator (Product #B0120100ML).

### 3.4. Protein Powder Analysis

The Kjeldahl method was used to obtain total protein content in blank and protein powder samples. Blank samples contained all reagents, but no protein powder, which permitted baseline zero-point correction. Endpoint titration was then used to calculate percent nitrogen, see Equation (1) [41]. The percent protein was calculated from the percent nitrogen calculation by multiplying by the corresponding conversion factor of 6.38 for milk and dairy, 6.25 for plant, and 6.07 for BSA [42,43].

(1) $$\%\;\mathrm{Nitrogen}=\left(\frac{\left(\mathrm{Standard}\;\mathrm{Acid}\;\left(\mathrm{mL}\right)-\mathrm{Blank}(\mathrm{mL}\right))\;*\;\left(\frac{0.001L}{1\mathrm{mL}}\right)\;*\;\left(N\;\mathrm{of}\;\mathrm{Acid}\right)\;*\;\left(\frac{14.007g}{\mathrm{mol}}\right)}{\mathrm{Weight}\;\mathrm{of}\;\mathrm{Sample}\;\left(g\right)}\right)*\;\left(100\right)$$

#### 3.4.1. Digestion

To each 250 mL digestion tube was added two Kjeldahl catalyst tablets, a sample of protein powder that was 0.5, 1.0, or 2.0 g and 5, 10, or 15 mL concentrated sulfuric acid. The tubes were place in a preheated (400 °C) block digester for 1 h 45 min, followed by cooling of the tubes to room temperature, and finally 40 mL of DI water was added to prevent precipitation.

#### 3.4.2. Distillation

To each distillation vial was added 20, 40, or 60 mL of 40% NaOH, depending on the amount of sulfuric acid used in the digestion step, and allowed to distill into 150 mL of 4% boric acid (with bromocresol green-methyl red mixed indicator), as the receiving solution. The mixed indicator gave the 4% boric acid solution a red color, which changed to green following distillation, indicating a rise in pH. The distillation time was set to 10 min for all trials.

#### 3.4.3. Titration

A 50 mL burette with 0.1 M hydrochloric acid was used to titrate each distilled sample. Titration was deemed finished when the color of final solution changed from green to pale pink, indicating that all ammonia in the solution was neutralized. The total amount of acid titrant required to neutralize the ammonia generated by distillation permitted the calculation of percent nitrogen from each protein powder sample using Equation (1). The percent nitrogen calculation was then multiplied by the respective conversion factor (6.38, 6.25, or 6.07), to calculate percent protein.

### 3.5. Ammonium Sulfate Chemical Check

Ammonium sulfate was used to test the distillation unit and reagents from the distillation step [44]. To a 250 mL Kjeldahl tube was added 2.0 g ammonium sulfate (99.99%), 75 mL DI water, 50 mL of 40% NaOH and the solution was distilled into 150 mL of 4% boric acid (with bromocresol green-methyl red mixed indicator), as the receiving solution. The resulting ammonium-borate complex was titrated as described above, and percent nitrogen was calculated as described above. A percent recovery was then calculated from the resulting percent nitrogen according to Equation (2).

(2) $$\%\;\mathrm{Recovery}=(\frac{\%\;\mathrm{Nitrogen}}{21.09})\;*\;\left(100\right)$$

## 4. Results

### 4.1. Kjeldahl Method Data

#### 4.1.1. Protein Powder Results

KM evaluation of protein powders provided the result that as sample size increased from 0.5 to 1.0 g, and finally 2.0 g, the percent nitrogen also increased to approach the theoretical maximum of 100% protein quantitation (Figure S1). The KM measurement was not as expected, since the percent nitrogen for a particular protein powder was anticipated be the same, regardless of the protein powder sample size.

Testing of 99.99% pure ammonium sulfate was used as a chemical check and verification of the distillation and titration steps. A 2.0 g sample of ammonium sulfate resulted in 99.99% recovery of ammonia. The 98.5% purity of commercially available L-lysine monohydrochloride was used to verify the digestion step and provided a theoretical maximum nitrogen content of 15%. Experimentally, the KM was used to determine the percent nitrogen values for lysine; the 0.5, 1.0, and 2.0 g samples calculated to 8.6, 12.3, and 14.7%, respectively, showing better agreement with the theoretical value (15%), as the sample size increased. Triplicate KM trials for both the ammonium sulfate and lysine, experimentally verified the 2.0 g sample consistently provided an accurate measure of true percent nitrogen.

**Figure 2:**
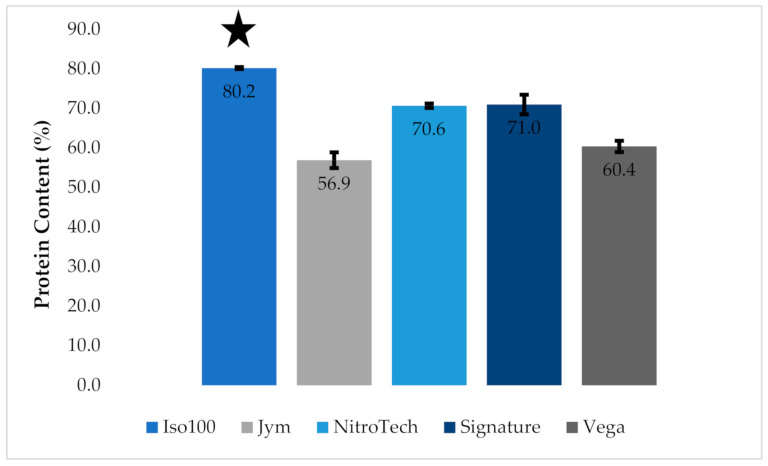
Bar graph of mean percent protein for five protein powder products, 2.0 g sample size. Star represents the significant difference between ISO100 and the other protein powders. Standard deviation is shown for each sample, where the error bar is based on a sampling size of 14 measurements (standard error of means (SEM)). 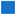 = ISO100 protein powder, 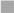 = JYM protein powder, 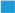 = NitroTech protein powder, 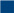 = Signature protein powder and 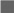 = Vega protein powder.

The KM determined protein content for 2.0 g samples, for five protein powders, based on 14 replicates, per product studied, see Figure 2.

The whey-based protein powder (JYM) and the plant-based protein powder (Vega) were found to contain the lowest protein content per 2.0 g sample, with the composition of protein in each product measured to be 56.9% and 60.4%, respectively. Three of the whey-based protein powders, NitroTech, Signature, and ISO100, were determined to have higher levels of protein, measured to be 70.6%, 71.0%, and 80.2%, respectively. When a one-way ANOVA test was performed on the five protein powders, a significant difference was found at the 0.05 critical alpha value with a *p*-value < 0.001. The ANOVA test confirmed there was a significant difference between the amount of protein in the ISO100 protein powder and the four other protein powders.

**Figure 3:**
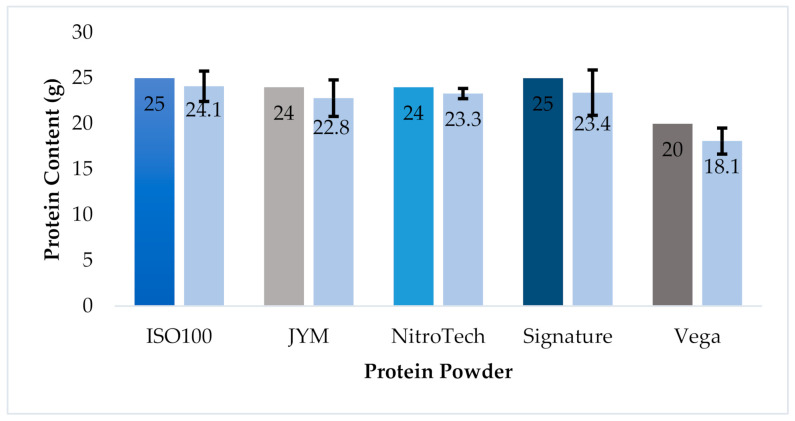
Bar graph comparing protein content as stated on product labels to experimental data from KM measurement. Standard deviation is shown for each product, with error bars based on a sampling size of 14 measurements (SEM).

The KM results for protein quantitation, using the 2.0 g protein powder sample size, were then compared to the information provided on the product label. According to the product label, a 30.0 g serving of ISO100 was expected to contain 25.0 g of protein. The KM result obtained for this product (80.2%) equated to a protein content of 24.1 g, or nearly 4% lower. This same analysis was repeated for each product (Table S1), with the same result that the KM quantitation provided a lower value for protein per serving than what was reported on the product labels, with differences ranging from 0.7–1.9 g. When the standard deviations were applied to the respective protein powders, there was no significant difference in protein content, between what was measured by the KM and what was reported on the labels, see Figure 3. A combination of all nitrogen contributors was taken into account and conversion factors were applied, product labels reflected the protein content that was determined by KM when the data from 14 trials were considered.

When the KM total protein values were used to calculate the amount of protein per container for each of the protein powders, the NitroTech protein powder contained the most protein with 722.3 g, because NitroTech has more servings per container out of all products tested, with 31 servings (Table S1). When the price of the products was considered, the whey-protein powder Signature was the best value out of the five protein powders, because this protein powder is the lowest priced protein powder of the products tested, at $20.24 for a two-pound container. When the cost of the container is divided by the protein per container, the cost of protein per container can be found. The whey-protein powder Signature is again the best value at $0.03 per gram of protein, because this protein powder has the most protein per container (631.8 g) at the lowest price ($20.24).

#### 4.1.2. Protein Spiking Results

**Figure 4:**
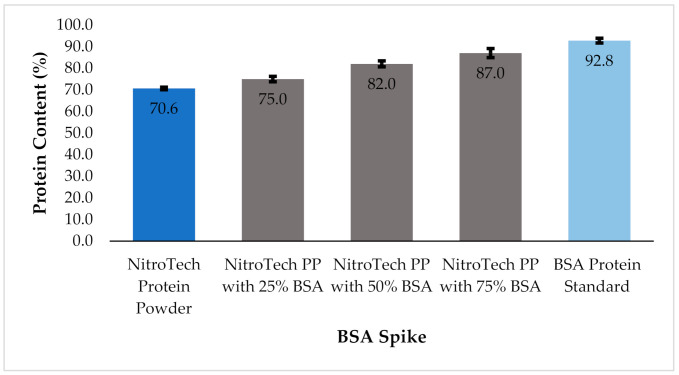
Bar graph showing percent protein values for 100% NitroTech protein powder (NitroTech PP) and each subsequent BSA spike (25%, 50%, and 75%), as well as the 100% BSA protein standard. Standard deviation is shown for each, error bar based on a sampling size of 5 measurements of a 2.0 g sample.

To test how product tampering, with a known protein, would affect the protein percent calculation, the whey-protein powder NitroTech was spiked with known amounts of BSA and analyzed using the KM. BSA made-up 25% (0.5 g), 50% (1.0 g), and 75% (1.5 g) of a 2.0 g sample, the other 75% (1.5 g), 50% (1.0 g), and 25% (0.5 g) was that of the NitroTech protein powder. When comparing the protein percentages of each; the NitroTech protein powder alone, the incremental spikes (25%, 50%, and 75%), and the BSA protein standard alone, the total protein content increases (Figure 4). The nitrogen content of a 2.0 g sample was calculated by multiplying the total nitrogen content by the respective amount of NitroTech and BSA used in the NitroTech:BSA spiked samples (25%, 50%, and 75%), resulting in two nitrogen calculations. Each nitrogen calculation was then multiplied by the appropriate conversion factor; 6.38 for the NitroTech protein powder and 6.07 for BSA, resulting in two protein totals. The two protein totals were added together, resulting in a final total protein content for a 2.0 g sample. The protein powder at 70.6% protein to 100% BSA, which was measured to be 92.8% protein. The BSA result is within the manufacturer specification of 90–100%.

### 4.2. Mid-Infrared (MIR) Spectroscopy Data

**Figure 5:**
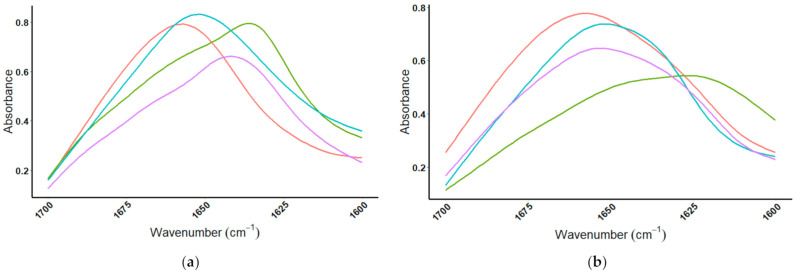
Amide I region (1700–1600 cm^−1^) of MIR spectrum for the eight protein standards: (**a**) whey proteins: 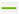 = β-lactoglobulin, 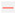 = α-lactalbumin, 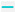 = BSA, 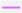 = IgG, and (**b**) non-whey proteins: 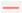 = brown rice, 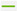 = casein, 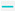 = egg albumin, and 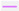 = pea.

MIR spectra for four whey proteins show the individual proteins can be differentiated from one another by analysis of the amide I region. When the amide I region is viewed, the individual proteins can be differentiated; Figure 5a shows the spectral overlay for the four whey protein standards, and Figure 5b displays the spectra of brown rice, casein, egg albumin, and pea proteins from 1700–1600 cm^−1^. The peak absorbance wavenumbers for the amide I region of the eight proteins are listed in Table 1.

The spectral overlay for the amide II region, from 1580–1500 cm^−1^, has been provided for the whey protein standards (Figure S2a), and brown rice, casein, egg albumin, and pea proteins (Figure S2b). The peaks in the amide II region are less discernable than the amide I region, however, the casein protein had an absorbance maximum that was distinct from the two plant proteins (brown rice and pea) and egg albumin at 1516 cm^−1^, and the whey protein, α-lactalbumin, had a noticeably different peak shape and maximum wavenumber (1541 cm^−1^) than the other three whey proteins (β-lactoglobulin, BSA, and IgG). When all milk proteins are compared to skim milk powder, casein has a dramatic effect on the overall amide I/II peaks, with skim milk having an amide I absorbance maximum at 1649 cm^−1^ and an amide II absorbance maximum at 1538 cm^−1^, see Figure S3.

Next, the lipid and carbohydrate signature regions of the MIR spectrum for each of the eight proteins were evaluated (brown rice, casein, egg albumin, pea, β-lactoglobulin, α-lactalbumin, BSA, and IgG) (Figure S4). When looking at the lipid peak, Figure S4a,b of the eight proteins, the pea protein was found to have a distinct lipid peak at 1743 cm^−1^, in contrast to the other seven proteins tested. When looking at the carbohydrate peak, Figure S4c,d shows that IgG, brown rice, casein, egg albumin, and pea proteins have a carbohydrate peak at the wavenumbers 1075, 1080, 1074, 1079, and 1082 cm^−1^, respectively, which is not observed for the other three dairy proteins. The exact wavenumbers for the lipid and carbohydrate peaks for brown rice and pea proteins are summarized in Table 1.

#### 4.2.1. Protein Powder Results

The MIR spectral overlays of the amide I/II regions of the five protein powders are shown in Figure S5. The amide I region of the five protein powders shows a common peak maximum at ≈1650 cm^−1^ (Figure S5a), but the magnitude of that absorbance maximum is lowest for the protein powder JYM (yellow) and highest for the protein powders ISO100 (red) and Signature (blue). The amide II region (Figure S5b) shows the same pattern as observed for the amide I, with the absorbance maxima being consistently at ≈1540 cm^−1^. The exact peak absorbance wavenumbers for each protein powder in the amide I/II regions are summarized in Table 2.

Given the similarity in the amide I and amide II peak regions across the protein powders, analysis of the lipid and carbohydrate spectral regions was reviewed. When the lipid and carbohydrate regions were reviewed, the lipid peak showed the most discernable distinction, Figure S6a shows the lipid peak region from 1770–1720 cm^−1^ for the five protein powders. The most noticeable peak observed, with the highest absorbance maximum, was that of JYM protein powder at 1744 cm^−1^. Absorbance for three other products was seen in this region (NitroTech, Signature, and Vega), but to a much lesser extent, having smaller absorbance maxima than the JYM protein powder. In the case of the ISO100 protein powder, no lipid peak was observed. When looking at the carbohydrate region for the five protein powders from 1150–1000 cm^−1^ (Figure S6b), all show low levels of absorbance around 1080 cm^−1^, but they are not distinct enough from one another for this wavenumber to be useful for differentiating these products. Tabulation of these result is summarized in Table 2.

**Figure 6:**
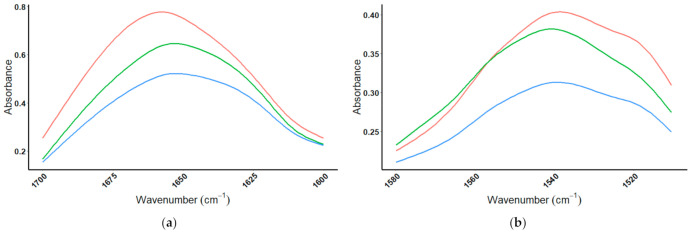
Amide I and amide II regions, comparing Vega protein powder to protein standards. MIR spectra of plant-based protein product (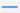 = Vega) and protein standards (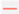 = Brown Rice and 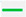 = Pea, observing (**a**) the amide I spectral region (1700–1600 cm^−1^), and (**b**) the amide II spectral region (1580–1510 cm^−1^).

To verify that the plant-based protein powder (Vega) was made-up of the two proteins listed on the product label, pea and brown rice, the amide I/II regions of the Vega protein powder were compared to the brown rice and pea protein standards (Figure 6). When comparing the amide I absorbance for all proteins to Vega; the amide I absorbance of the Vega protein powder closely resembles that of pea protein standard consistent with pea protein being the major protein constituent of Vega (Figure 6a). Inspection of the amide II spectral overlay (Figure 6b) shows that the Vega protein powder contains absorbance characteristics consistent with the brown rice protein standard. When both the amide I and amide II regions of the Vega protein powder are considered, the results are consistent with pea and brown rice being the two protein contributors. The wavenumber for the amide I peak for the Vega protein powder and the brown rice and pea protein standards were all around 1652 cm^−1^, while the amide II peak for all three was 1540 cm^−1^, as seen in Table 3.

**Figure 7:**
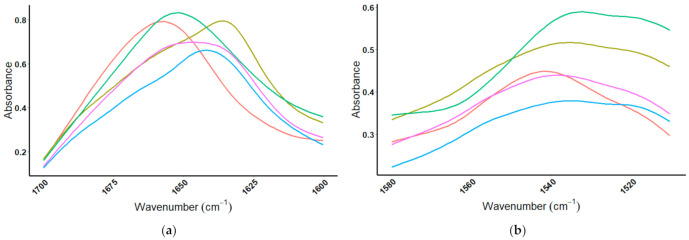
Amide I and amide II regions comparing ISO100 protein powder to protein standards. MIR spectrum of the four whey protein standards (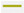 = β-lactoglobulin, 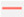 = α-lactalbumin, 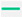 = BSA, 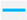 = IgG), and whey protein product 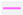 = ISO100, in (**a**) the amide I spectral region (1700–1600 cm^−1^), and (**b**) the amide II spectral region (1580–1510 cm^−1^).

The MIR spectra for whey protein standards (β-lactoglobulin, α-lactalbumin, BSA, and IgG) were then compared to ISO100. The protein powder ISO100 was selected for this comparison because it did not show absorbance in the lipid or carbohydrate regions, indicating that the composition of the mixture may be entirely whey proteins. The amide I/II regions are shown in Figure 7. The amide I absorbance maximum for ISO100 at 1646 cm^−1^ does not match perfectly to any one of the whey protein standards (Figure 7a), which was to be expected considering the product label lists the protein ingredients as consisting of two contributors, hydrolyzed whey protein isolate and whey protein isolate. The amide II absorbance maximum for ISO100 at 1539 cm^−1^ again does not match to any one protein, but is the result of a mixture (Figure 7b). When both the amide I/II peaks are analyzed, the spectrum of the protein powder ISO100 is consistent with a combination of the whey protein components (β-lactoglobulin, α-lactalbumin, BSA, and IgG), which are expected to be in the product. The amide I/II wavenumbers of maximum amplitude absorbance (1646 cm^−1^ and 1539 cm^−1^) are unique from the four whey proteins, but in the middle of the grouping. The wavenumbers for the amide I/II peaks for the whey protein standards and the protein powder ISO100 are summarized in Table 4.

**Figure 8:**
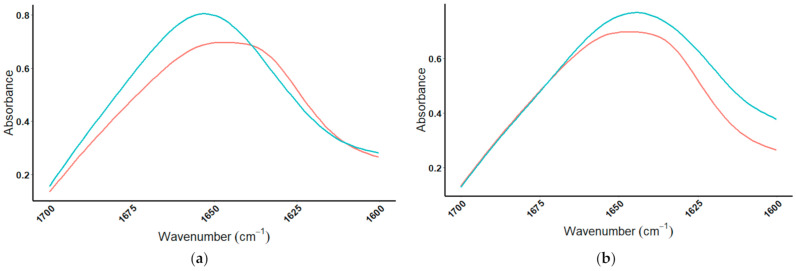

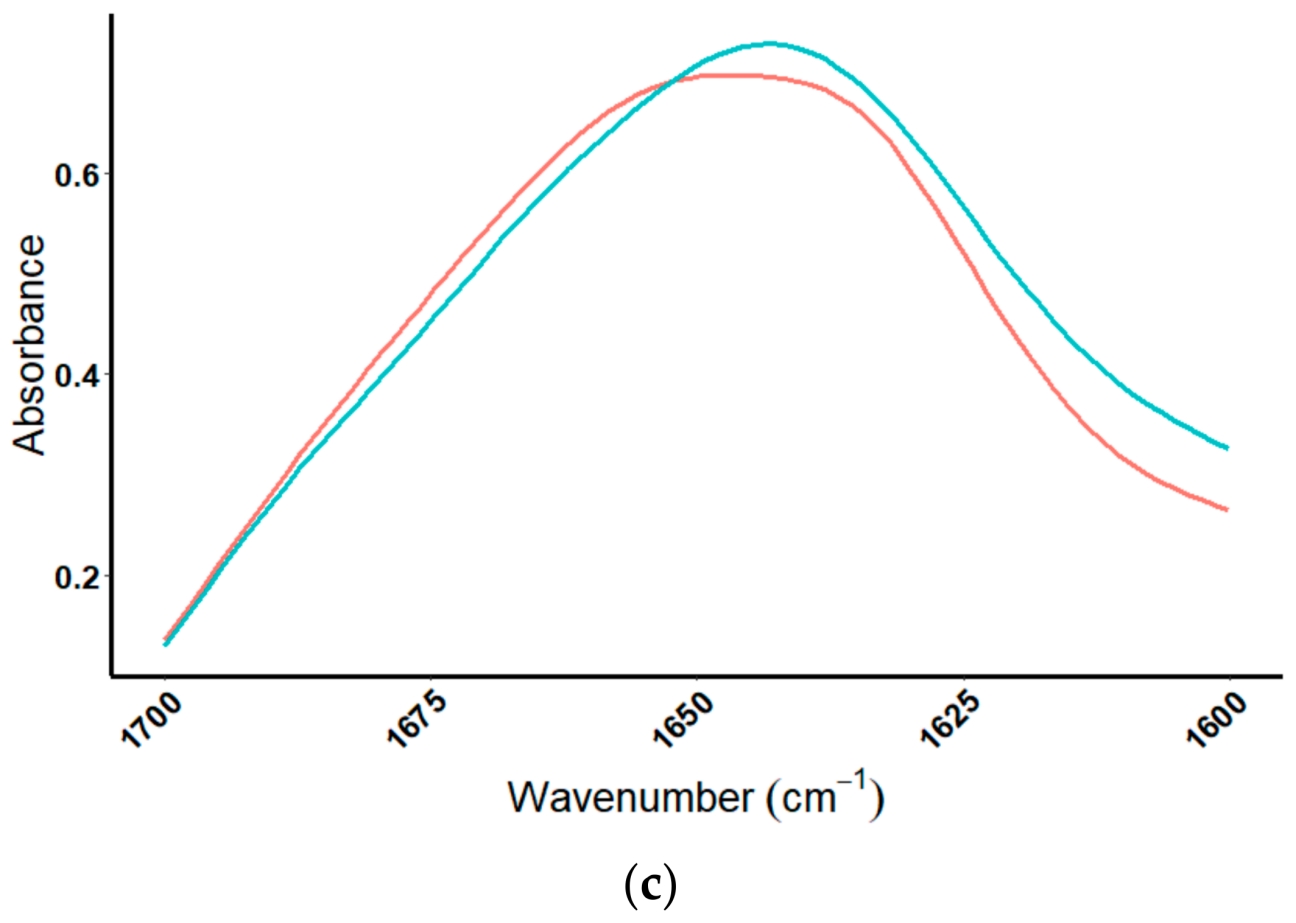
Amide I region comparing ISO100 protein powder to known mixtures of protein standards. (**a**) MIR spectrum comparing ISO100 protein powder to a (1:1) mixture of α-lactalbumin:β-lactoglobulin. (**b**) MIR spectrum comparing ISO100 protein powder to a (1:1:1) mixture of α-lactalbumin:β-lactoglobulin:BSA. (**c**) MIR spectrum comparing ISO100 protein powder to a (1:1:1:1) mixture of α-lactalbumin:β-lactoglobulin:BSA:IgG. In all cases, 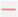 = ISO100 protein powder and 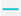 = mixture of proteins.

The whey protein standards were then mixed in a 1:1 (mass/mass) ratio and compared to the ISO100 protein powder. The MIR spectra of the mixtures are seen in Figure 8, specifically looking at the amide I region, comparing the ISO100 protein powder to three different protein mixtures. As each protein is added, the spectra of the mixture and that of the protein powder line-up better; Figure 8a compares the ISO100 protein powder to a mixture of β-lactoglobulin and α-lactalbumin, Figure 8b compares the ISO100 protein powder to a mixture of β-lactoglobulin, α-lactalbumin, and BSA, and finally Figure 8c compares the ISO100 protein powder to a mixture of β-lactoglobulin, α-lactalbumin, BSA, and IgG. When comparing the ISO100 protein powder to the protein standard mixtures, the one that most closely resembles the protein powder is the mixture that contains all four of the protein standards; Figure 8c.

The whey protein product JYM, which is a “protein blend” consisting of whey protein isolate, micellar casein, milk protein isolate, and egg protein was compared to the MIR spectra for each of the constituents. The product label states that 50% of the 24 g of protein per serving is derived from casein protein, 40% whey protein, and 10% egg protein. To begin, the MIR spectrum of JYM was compared directly to the main protein constituent, casein. The MIR spectra for the amide I peak of JYM and casein are shown in Figure S7a, JYM has an absorbance maximum at 1652 cm^−1^, and casein at 1627 cm^−1^. The absorbance peak shape was also distinctly different between the two, with JYM being more uniform, and casein being broader and more intense. Next, the amide I peak of JYM was compared to that of the four whey protein standards (see Figure S7b). From the amide I peak amplitude and signal broadness, it may be predicted that the primary protein that makes-up the JYM protein powder is not one or a combination of the four whey proteins. The amide I peak of JYM is broad and shallow, while the four whey protein peaks are distinct in peak amplitude, absorbance wavenumber, and more intense with respect to magnitude of absorbance. The ingredient label listed egg protein as a component of the protein blend, so the MIR spectrum for egg albumin was compared to JYM. The overlay of MIR spectra for JYM and the egg albumin protein standard are shown in Figure S7a. While, the amide I peak of JYM is broad and shallow and the amide I peak of the egg albumin standard has a distinct peak amplitude, both have a maximum absorbance at 1652 cm^−1^. The lipid peak was also evaluated, comparing JYM to that of the casein, whey, and egg albumin protein standards (see Figure S7d). The figure shows that while the JYM protein powder has a very distinct peak in this region, none of the protein standards have a peak in this region. Inspection of the JYM protein powder label listed coconut oil as an ingredient, and it is thought that the observed lipid peak is due to the coconut oil.

#### 4.2.2. Protein Spiking Results

**Figure 9:**
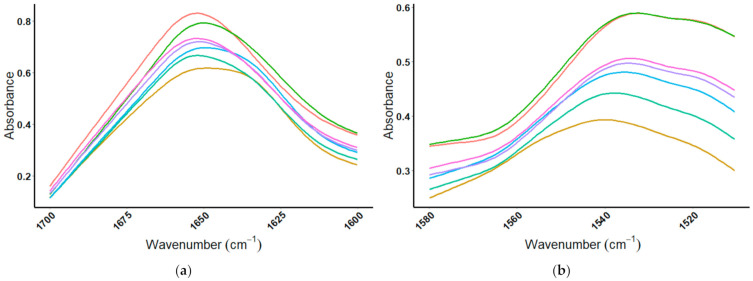
Amide I and amide II regions of NitroTech protein powder, bovine serum albumin (BSA), and spikes. MIR spectra showing the doping of NitroTech protein powder with BSA to a ratio of 1:10 (%m/m), where (**a**) is the amide I spectral region (1700–1600 cm^−1^), and (**b**) is the amide II spectral region (1580–1510 cm^−1^). 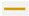 = NitroTech protein powder, 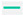 = NitroTech:BSA(1:2), 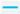 = NitroTech:BSA(1:4), 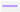 = NitroTech:BSA(1:6), 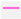 = NitroTech:BSA(1:8), 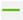 = NitroTech:BSA(1:10), 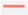 = BSA.

NitroTech was spiked with increasing amounts of BSA, using a percent mass/mass ratio, and the amide II peak was monitored as it shifted from 1540 to 1532 cm^−1^ at a final ratio of 1:10 NitroTech:BSA. The exact amide II peak absorbance wavenumbers for NitroTech, BSA, and subsequent spiked samples are summarized in Table 5. The amide I peak of NitroTech (brown; bottom), BSA (red; top), and the BSA-spiked samples (cyan-green) are shown in Figure 9a. While NitroTech and BSA have amide I peaks consistently around 1650 cm^−1^, a general trend could be seen; as the protein powder NitroTech was spike with increasing amounts of BSA, the peak shape changes from the broader peak of NitroTech to the more pronounced peak of BSA. The amide II peak of NitroTech, BSA, and the subsequent spiked samples of NitroTech with BSA are shown in Figure 9b. The amide II peak of NitroTech can be seen to shift to a lower wavenumber and align with the shape of the BSA with each successive addition of BSA.

Given an observable trend for the amide II absorbance for NitroTech, with increasing amounts of BSA, the lipid and carbohydrate regions of the MIR spectra were reviewed. NitroTech has a lipid peak at about 1743 cm^−1^, which gradually disappears as the ratio of BSA increases from 1:1 to 1:10 (see Figure S8a). The carbohydrate region (1150–1000 cm^−1^) showed little discernable variation upon product doping (Figure S8b). The absorbance wavenumbers for NitroTech, BSA, and subsequent ratios of the two are summarized in Table 5.

#### 4.2.3. Amino Acid Spiking Results

**Figure 10:**
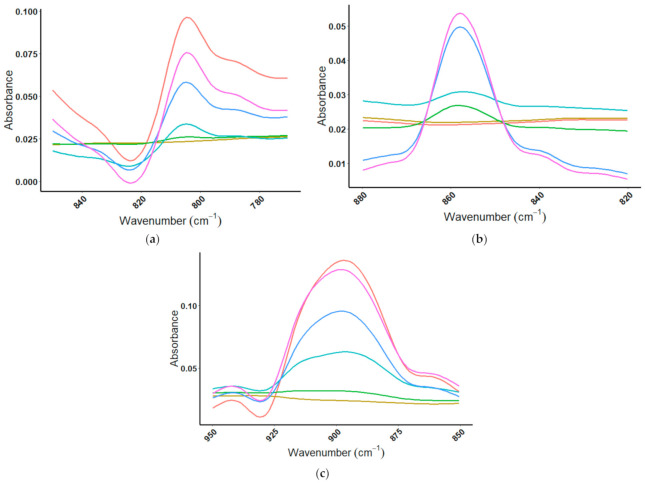
MIR spectral overlays for ISO100 with increasing amount of amino acid to a ratio of 1:3. (**a**) Doping with the amino acid glutamic acid spike in the range of 850–770 cm^−1^ (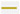 = ISO100, 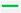 = ISO100:GA (10:1), 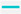 = ISO100:GA (4:1), 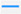 = ISO100:GA (1:1), 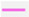 = ISO100:GA (1:3), 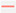 = GA); (**b**) doping with the amino acid lysine over the range from 880–820 cm^−1^ (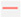 = ISO100, 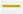 = ISO100:Lysine (10:1), 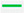 = ISO100/Lysine (4:1), 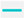 = ISO100:Lysine (1:1), 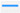 = ISO100:Lysine (1:3), 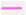 = Lysine); and (**c**) doping with the amino acid glycine over the range from 950–850 cm^−1^ (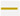 = ISO100, 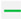 = ISO100:Glycine (10:1), 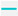 = ISO100:Glycine (4:1), 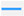 = ISO100/Glycine (1:1), 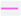 = ISO100/Glycine (1:3), 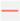 = Glycine).

Spiking of a protein powder with the protein BSA was able to be visualized by MIR spectroscopy, leading to an investigation as to whether MIR spectra may be used to identify amino acid doping of commercial protein powder products. In the case of amino acid spiked protein powders, the fingerprint region (1200–700 cm^−1^) of the MIR spectrum was studied. When ISO100 was spiked with increasing amounts of glutamic acid, a discernable peak appeared at 806 cm^−1^, where there was none in the protein powder (Figure 10a). The same trend was seen with lysine (Figure 10b), that as ISO100 was spiked with increasing amounts of the amino acid lysine, the appearance of a distinct peak could be observed at 857 cm^−1^. The protein powder, ISO100 has no peak at 857 cm^−1^, while lysine has a very distinctive peak. In Figure 10c, the MIR spectral overlays from 950–850 cm^−1^ for ISO100 and ISO100 with added glycine, show a peak attributable to glycine at 909 cm^−1^, where there was none in the protein powder. The amino acids glutamic acid and glycine begin to be observed with as little as 10% (ISO100/amino acid) concentration, with a discernable peak visible at the 25% (ISO100/amino acid) concentration. The amino acid lysine was observed at doping levels of 25% (ISO100/amino acid) concentration.

## 5. Discussion

In this study, the Kjeldahl method was used to survey the protein content of five commercially available protein powders and the amino acid lysine. The KM took approximately five hours for sample analysis, in contrast, the mid-infrared spectroscopy analysis of four whey proteins could be performed in approximately ten minutes per sample, and analysis of amide I and amide II regions of the MIR spectrum permitted qualitative differentiation between the proteins studied. Expanding the MIR analysis to brown rice, egg albumin, and pea protein led to the observation that spectral regions associated with lipid and carbohydrate absorption could be used as signatures for these proteins that were not present for the dairy proteins.

MIR analysis was also used for qualitative analysis, the amide I/II MIR spectral comparison reveled that the one plant-based protein powder analyzed contained the proteins that were stated on the label; pea and brown rice. By looking at the peak shape, qualitatively it was found that the amide I/II regions can be used to identify the individual whey proteins in a mixture, each protein has a distinct signature at a specific wavenumber and peak shape in these regions, and these characteristics can be used to identify the protein.

MIR analysis of protein powder intentionally adulterated with either protein (e.g., BSA) or amino acids (e.g., lysine, glutamic acid, glycine) provided evidence that product tampering could be readily monitored by this method. Through the KM, protein spiking of the protein powder NitroTech with increasing amounts of BSA resulted in an increase in total protein content. Amide I/II MIR spectral comparisons of the NitroTech protein powder and NitroTech:BSA spiked samples showed that as BSA amounts were increased, a change in peak shape was visualized. Amino acid spiking experiments revealed that MIR can be used to detect and visualize amino acid spiking in protein powders. For the three amino acids used in this study (glutamic acid, lysine, and glycine), the fingerprint region (wavenumbers 1200–700 cm^−1^) was used for structural confirmation and was able to differentiate between the amino acids. As product doping with amino acids has been documented, the threshold of detection for MIR was estimated to be between 10–25% for the three amino acids studied. The current investigation provides a framework for the development of a quantitative approach when looking at amino acid product tampering by MIR spectroscopy. The next study beyond amino acid analysis will be the quantification of the whey proteins β-lactoglobulin, α-lactalbumin, BSA, and IgG using MIR.

## 6. Conclusions

The work detailed demonstrates the potential to use MIR spectroscopy for quality control and quality assurance, when combined with the KM, in the testing of dietary supplements such as whey protein powders. Evidence was presented that MIR spectroscopy is useful for qualitative protein analysis, because this method can differentiate individual protein components commonly used in protein powders, and MIR can also be used to detect product adulteration by either proteins or amino acids.

## Figures and Tables

**Table 1:** MIR data for proteins in the amide I/II, lipid, and carbohydrate spectral regions.

Protein Standard	Amide I (cm^−1^)	Amide II (cm^−1^)	Lipid (cm^−1^)	Carbohydrate (cm^−1^)
β-lactoglobulin	1635 ± 1	1537 ± 2	N/A	N/A
α-lactalbumin	1657 ± 5	1541 ± 2	N/A	N/A
BSA	1651 ± 1	1528 ± 4	N/A	N/A
IgG	1642 ± 4	1540 ± 1	N/A	1075 ± 1 (w) *
Casein	1627 ± 1	1516 ± 0	N/A	1074 ± 0 (w) *
Egg Albumin	1652 ± 0	1539 ± 0	N/A	1079 ± 0 (w) *
Brown Rice	1653 ± 0	1539 ± 0	N/A	1080 ± 0 (w) *
Pea	1653 ± 0	1541 ± 1	1743 ± 0 (w)	1082 ± 0 (m) *

* Lipid/carbohydrate peak abbreviations: m = medium and w = weak absorbance.

**Table 2:** MIR data of protein powders in the amide I/II, lipid, and carbohydrate spectral regions.

Protein Powder	Amide I (cm^−1^)	Amide II (cm^−1^)	Lipid (cm^−1^)	Carbohydrate (cm^−1^)
ISO100	1646 ± 0	1539 ± 0	N/A	1079 ± 0
JYM	1652 ± 0	1539 ± 0	1745 ± 1 (m) *	1080 ± 0
NitroTech	1652 ± 0	1540 ± 0	1743 ± 0 (w) *	1079 ± 0
Signature	1645 ± 0	1539 ± 0	1742 ± 0 (w) *	1078 ± 0
Vega	1652 ± 0	1539 ± 0	1741 ± 0 (w) *	1079 ± 1

* Lipid peak abbreviations: N/A = Not Applicable, m = medium and w = weak absorbance.

**Table 3:** IR data comparing brown rice and pea protein standards to Vega protein powder.

Protein	Amide I (cm^−1^)	Amide II (cm^−1^)
Brown Rice	1653 ± 0	1539 ± 0
Pea	1653 ± 0	1541 ± 1
Vega protein powder	1652 ± 0	1539 ± 0

**Table 4:** IR data comparing whey protein standards to ISO100 protein powder.

Protein	Amide I (cm^−1^)	Amide II (cm^−1^)
β-lactoglobulin	1635 ± 1	1537 ± 2
α-lactalbumin	1657 ± 5	1541 ± 2
BSA	1651 ± 1	1528 ± 4
IgG	1642 ± 4	1540 ± 1
ISO100 protein powder	1646 ± 0	1539 ± 0

**Table 5:** IR data of whey protein powder NitroTech, spiked with a known amount of a single whey protein, bovine serum albumin (BSA).

Spike Ratio	Amide I (cm^−1^)	Amide II (cm^−1^)	Lipid (cm^−1^)	Carbohydrate (cm^−1^)
NitroTech PP	1652 ± 0	1540 ± 0	1743 ± 0	1079 ± 0
NitroTech/BSA(1:2)	1652 ± 0	1539 ± 0	1742 ± 0	1080 ± 1
NitroTech/BSA(1:4)	1652 ± 1	1537 ± 3	1743 ± 1	1081 ± 2
NitroTech/BSA(1:6)	1651 ± 1	1532 ± 1	1743 ± 1	1082 ± 0
NitroTech/BSA(1:8)	1650 ± 3	1531 ± 4	1743 ± 4	1082 ± 2
NitroTech/BSA(1:10)	1651 ± 1	1532 ± 1	1743 ± 0	N/A *
BSA Protein	1651 ± 1	1528 ± 4	N/A *	N/A *

* Lipid peak abbreviations: N/A = Not Applicable.

## Data Availability

The data presented in this study are available in the supplemental materials and the thesis of Rose Saxton of title “Whey protein powder analysis by Kjeldahl and mid-infrared spectroscopy”.

## Supplementary Materials

The following are available online at https://www.mdpi.com/article/10.3390/foods10051033/s1, Figure S1: Bar graph of mean percent nitrogen for five protein powder products, at three sample sizes: 0.5, 1.0, and 2.0 g, Table S1: Protein content of protein powders, comparing label to Kjeldahl method results, Figure S2: Amide II region of MIR spectrum for all protein standards, Figure S3: Amide I/II region of MIR spectrum for all milk protein standards and skim milk powder, Figure S4: Lipid and carbohydrate regions for all protein standards, Figure S5: Amide I and amide II regions of protein powders, Figure S6: Lipid and carbohydrate region of protein powders, Figure S7: Amide I and lipid regions comparing JYM protein powder to protein standards, Figure S8: Lipid and carbohydrate regions of NitroTech protein powder, BSA, and spikes.
