# Supplementary material for: Whey Protein Powder Analysis by Mid-Infrared Spectroscopy
Source: Foods. 2021 May 10;10(5):1033. doi: 10.3390/foods10051033 (PMC8151012; doi:10.3390/foods10051033)
Supplement: Supplementary file 1 [file foods-10-01033-s001.zip › foods-1191597-supplementary.pdf]

Supplementary Material

Figure S1 shows a bar graph, showing total percent nitrogen, for each of the five protein powders tested at the three different sample amounts tested (0.5 g, 1.0 g and 2.0 g). From Figure S1 it can be seen that for each of the protein powders tested, as sample size is increased, percent nitrogen also increases. The figure also shows when the standard deviations for each protein powder are applied, each sample size is distinctly different from one another, as to the total percent nitrogen found using the Kjeldahl Method.

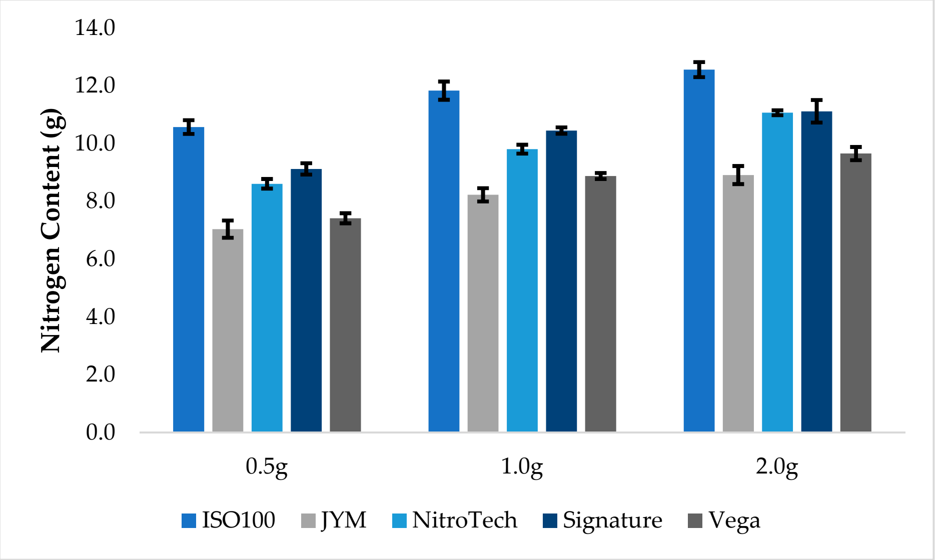

Figure S1. Bar graph of mean percent nitrogen for five protein powder products, at three sample sizes: 0.5 g, 1.0 g and 2.0 g. Standard deviation are shown for each, error bar based on a sampling size of 14 measurements (means with Standard Error of Means (SEM)). ■ = ISO100 protein powder, ■ = JYM protein powder, ■ = NitroTech protein powder, ■ = Signature protein powder and ■ = Vega protein powder.

Table S1 shows serving size (g), servings per container (g), and protein per serving (g) for each of the protein powders tested, all of which came directly from product labeling. Table S1 also shows protein per serving (g) and the standard deviations, that were found experimentally using the Kjeldahl Method, for each protein powder tested. The experimental protein per serving (g) was then used to find protein per container (g) and the value of the protein per container (g). From Table S1 it can be seen that the plant-based protein powder was found to contain the least amount of protein per serving (g), out of the five protein powders tested; both from product labeling and experimentally. Table S1 shows that out of the four whey protein powders tested, ISO100 contains the most protein per serving (g), with 24 g. The whey-based protein powder, NitroTech, contains the most protein per container (g) with 722 g and the whey-based protein powder, Signature, is the best valued protein powder.

Table S1: Protein content of protein powders, comparing label to Kjeldahl Method results.

Protein Powder	Serving Size (g)	Servings per Container (#)	Label-Protein per Serving (g)	KM-Protein per Serving (g)	Protein per Container (g)	Cost of Container ($)	Value of Protein per Container ($)
**ISO100**	30.0	24.0	25.0	24.1 ± 1.7	578.4	$32.99/ 1.6 pounds	$0.06
**JYM**	40.0	23.0	24.0	22.8 ± 2.0	524.4	$34.99/ 2.0 pounds	$0.07
**NitroTech**	33.0	31.0	24.0	23.3 ± 0.6	722.3	$32.99/ 2.2 pounds	$0.05
**Signature**	33.0	27.0	25.0	23.4 ± 2.5	631.8	$20.24/ 2.0 pounds	$0.03
**Vega**	30.0	18.0	20.0	18.1 ± 1.4	325.8	$29.99/ 1.3 pounds	$0.09

Figure S2 shows the mid-infrared spectrum; showing the amide II region (1580-1500 cm^{-1}), for all eight of the protein standards tested. From Figure S2 a, it can be seen that the protein standard α-lactalbumin (1541 cm^{-1}) has a distinctly different maximum absorbance then the other whey protein standards. From Figure S2 b, it can be seen that the protein standard casein (1516 cm^{-1}) has a distinctly different absorbance maximum from the other protein standards.

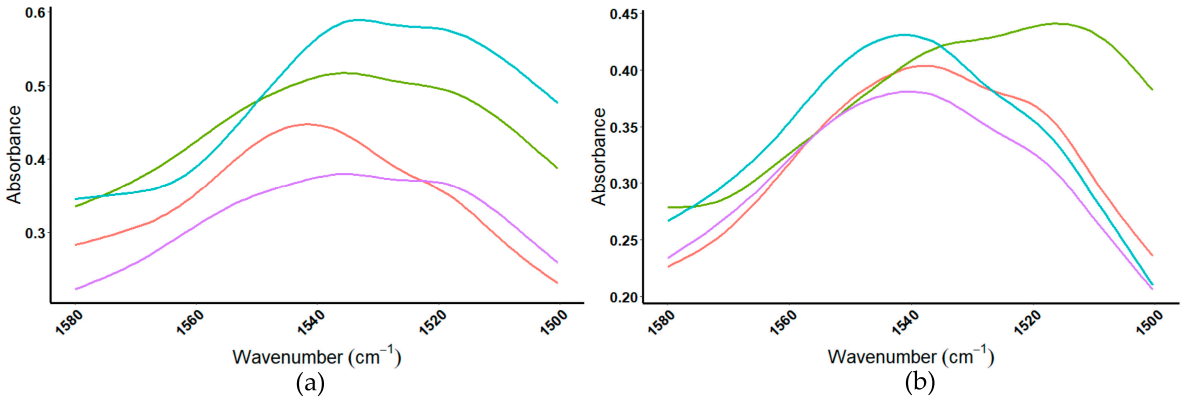

Figure S2. Amide II region of MIR spectrum for all protein standards. (a) MIR spectra of the whey protein standards — = β-lactoglobulin, — = α-lactalbumin, — = BSA, — = IgG, comparing the amide II spectral region (1580-1500 cm^{-1}), and (b) — = Brown Rice, — = Casein, — = Egg Albumin, — = Pea, comparing the amide II spectral region (1580-1500 cm^{-1}).

Figure S3 shows the mid-infrared spectrum; showing the amide I/II regions (1700-1500 cm^{-1}), for each of the five milk proteins tested and that of skim milk powder. From Figure S3, it can be seen that when all milk proteins are compared to skim milk powder, casein has a dramatic effect on the overall amide I/II peaks; with skim milk having an amide I absorbance maximum at 1649 cm^{-1} and an amide II absorbance maximum at 1538 cm^{-1}.

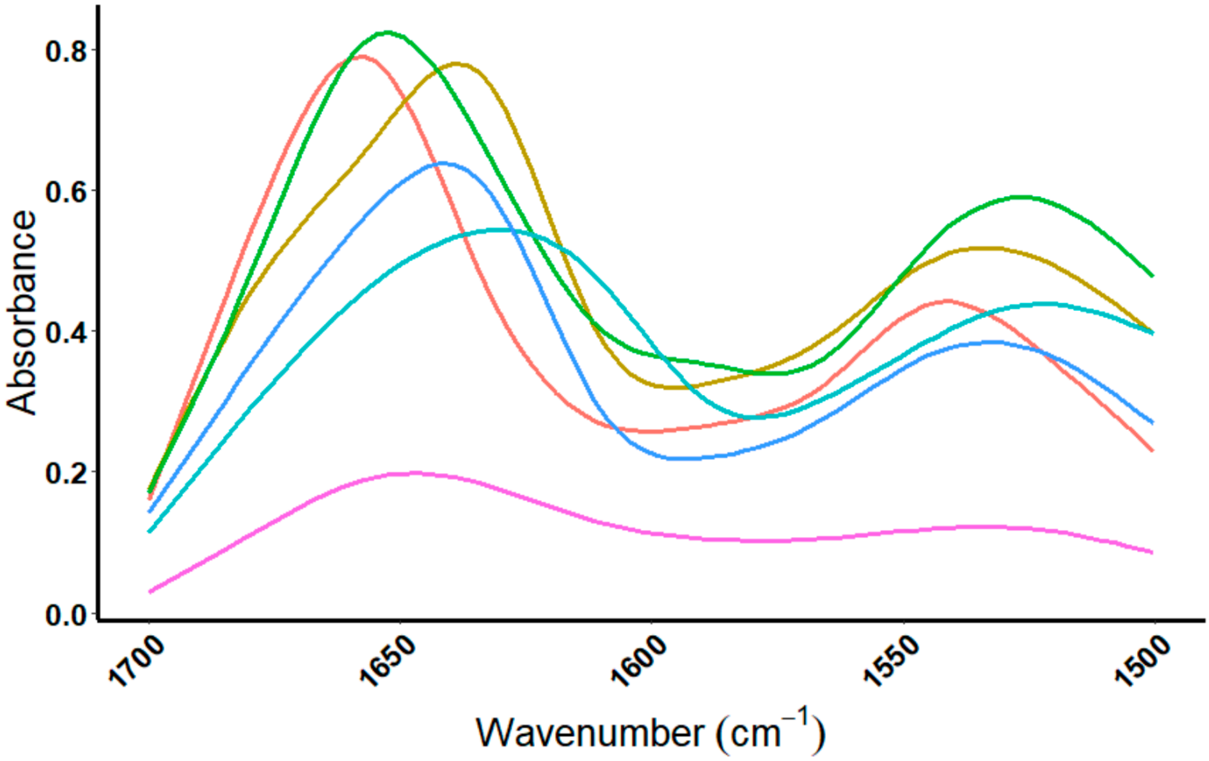

Figure S3. Amide I/II region of MIR spectrum for all milk protein standards and skim milk powder. MIR spectra of the whey protein standards —= β-lactoglobulin, — = α-lactalbumin, — = BSA, — = IgG, — = casein, and — = skim milk powder, comparing the amide I/II spectral regions (1700-1500 cm^{-1}).

Figure S4 shows the mid-infrared spectrum; showing the lipid (1740 cm^{-1}) and carbohydrate (1080 cm^{-1}) regions, for each of the eight protein standards tested. From Figure S4 a, it can be seen that when all the whey protein are compared to each other, none of them have a peak in the lipid region. From Figure S4 b, it can be seen that when the other protein standards are compared to each other, pea has a distinct peak in the lipid region at 1743 cm^{-1}. From Figure S4 c, it can be seen that when all the whey protein standards are compared to each other, IgG has a broad peak in the carbohydrate region at 1075 cm^{-1}. From Figure S4 d, it can be seen that when the other protein standards are compared to each other; brown rice, casein, egg albumin, and pea all have peaks in the carbohydrate region at 1080 cm^{-1}, 1074 cm^{-1}, 1079 cm^{-1}, and 1082 cm^{-1} respectively.

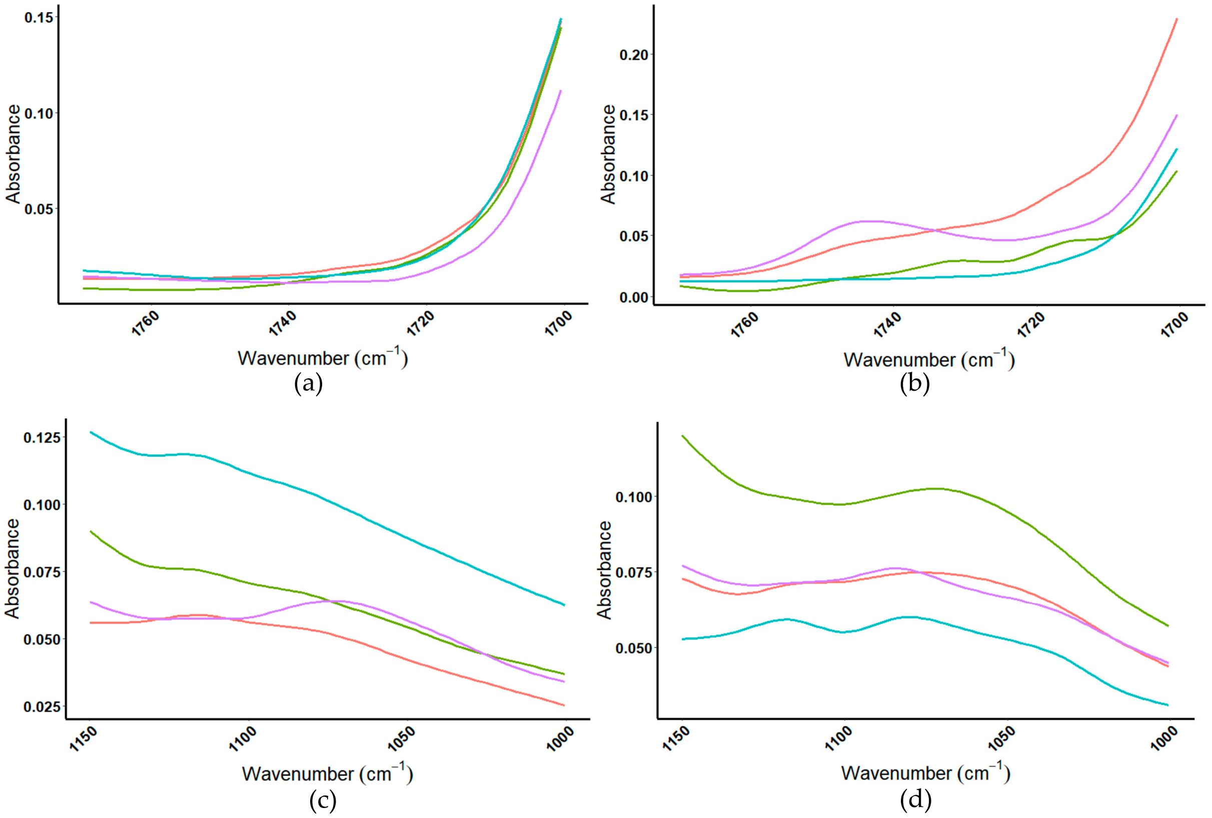

Figure S4. Lipid and carbohydrate regions for all protein standards. (a) MIR spectra of the whey proteins = β-lactoglobulin, = α-lactalbumin, = BSA, = IgG, comparing the lipid peak spectral region (≈1743 cm^{-1}), (b) MIR spectra of = Brown Rice, = Casein, = Egg Albumin, = Pea, comparing the lipid peak spectral region (≈1743 cm^{-1}), (c) MIR spectra of the whey proteins, = β-lactoglobulin, = α-lactalbumin, = BSA, = IgG, comparing the carbohydrate peak spectral region (≈1080 cm^{-1}), and (d) MIR spectra of = Brown Rice, = Casein, = Egg Albumin, = Pea, comparing the carbohydrate peak spectral region (≈1080 cm^{-1}).

Figure S5 shows the mid-infrared spectrum; showing the amide I/II regions (1700-1510 cm^{-1}), for all five of the protein powders tested. From Figure S5 a, it can be seen that the amide I region of all five protein powders have an absorbance maximum around 1650 cm^{-1}; with the protein powder JYM having the lowest. From Figure S2 b, it can be seen that the amide II region of all five protein powders have an absorbance maximum at 1540 cm^{-1}; again, the protein powder JYM has the lowest absorbance.

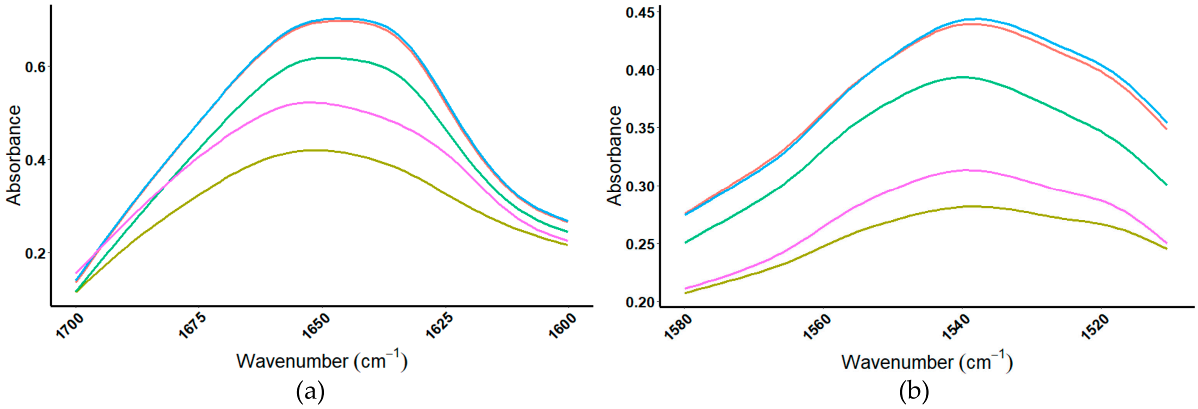

Figure S5. Amide I and amide II regions of protein powders. (a) MIR spectra of four whey protein powders and one pea protein powder in the amide I spectral region (1700-1600 cm^{-1}), and (b) the amide II spectral region (1580-1510 cm^{-1}), where = ISO100, = JYM, = NitroTech, = Signature, = Vega.

Figure S6 shows the mid-infrared spectrum; showing the lipid (1740 cm^{-1}) and carbohydrate (1080 cm^{-1}) regions, for each of the protein powders tested. From Figure S6 a, it can be seen that the protein powder JYM has a significantly higher maximum absorbance in the lipid region at 1745 cm^{-1}, when compared to the other protein powders. From Figure S6 b, it can be seen that all five of the protein powders tested have peaks in the carbohydrate region around 1080 cm^{-1}.

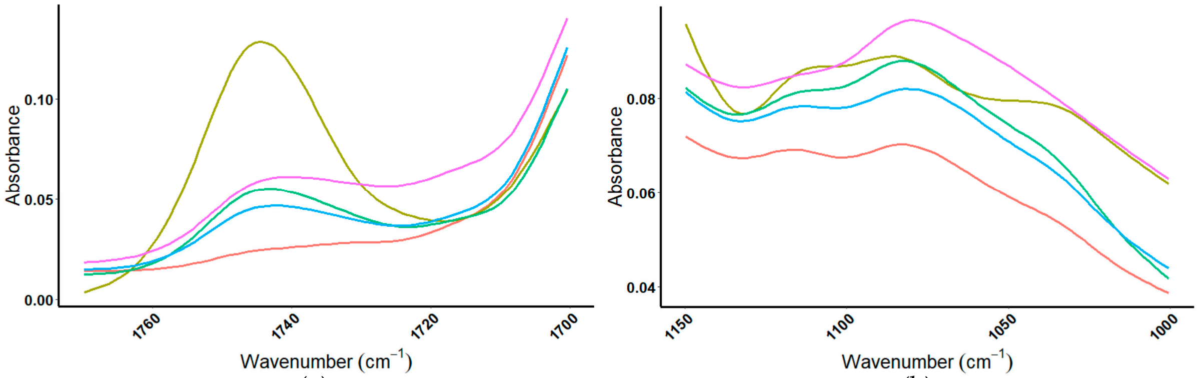

(a) (b)

Figure S6. Lipid and carbohydrate region of protein powders. (a) the lipid spectral region (≈1743 cm^{-1}), and (b) the carbohydrate spectral region (≈1080 cm^{-1}) for = ISO100, = JYM, = NitroTech, = Signature, = Vega.

Figure S7 shows the mid-infrared spectrum; showing the amide I (1700-1600 cm^{-1}) and lipid (1740 cm^{-1}) regions, comparing the protein powder JYM to proteins stated on the label. From Figure S7 a, it can be seen that the protein powder JYM and the protein standard casein have distinctly different amide I peaks; with JYM having a maximum absorbance at 1652 cm^{-1} and casein at 1627 cm^{-1}. From Figure S7 b, it can be seen that the protein powder JYM and the four whey protein standards have distinctly different amide I peaks; with JYM having a maximum absorbance at 1652 cm^{-1} and β-lactoglobulin at 1635 cm^{-1}, α-lactalbumin at 1657 cm^{-1}, BSA at 1651 cm^{-1}, and IgG at 1642 cm^{-1}. From Figure S7 c, while it can be seen that the protein powder JYM and the protein standard egg albumin have distinctly different amide I peak absorbances, both have a maximum absorbance at 1652 cm^{-1}. From Figure S7 d, it can be seen that when the six protein standards and the protein powder JYM are compared, only JYM has a lipid peak at 1745 cm^{-1}.

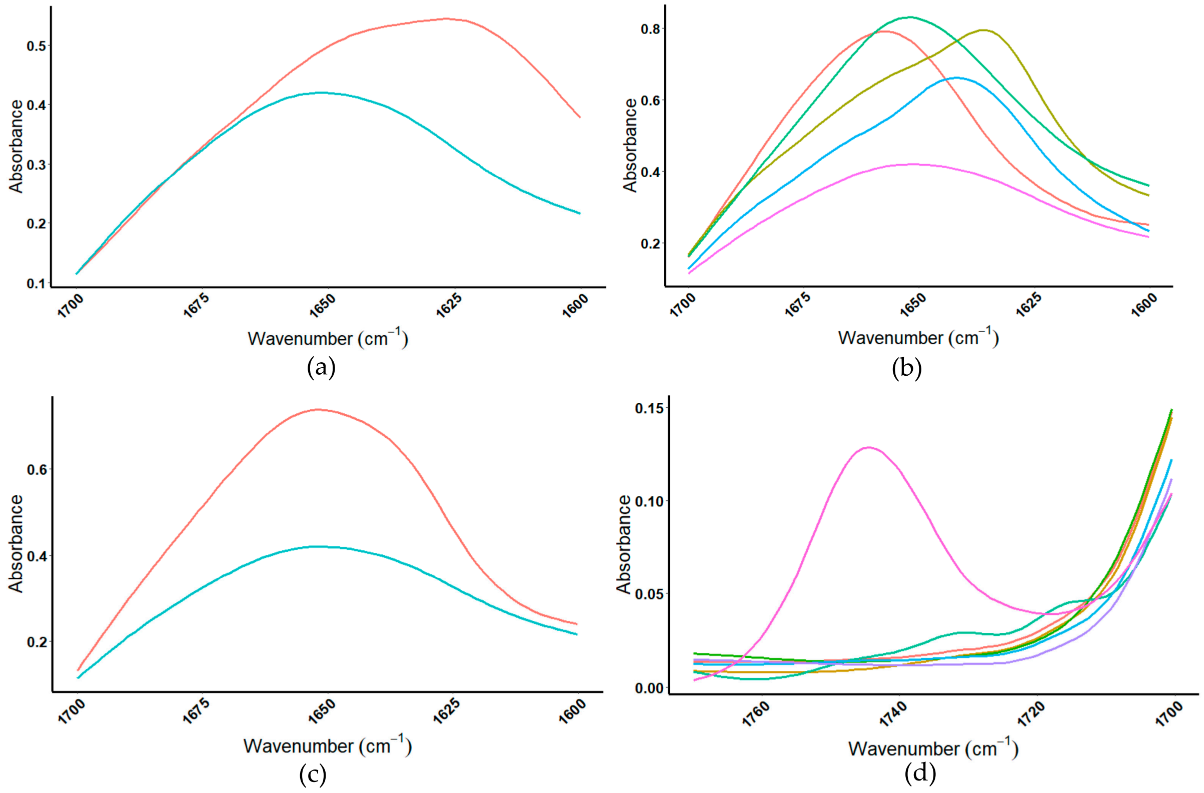

Figure S7. Amide I and lipid regions comparing JYM protein powder to protein standards. MIR spectra of protein product JYM, where (a) is the amide I spectral region (1700-1600 cm^{-1}) of = JYM and = casein, (b) is the amide I spectral region of the four whey protein standards = β-lactoglobulin, = α-lactalbumin, = BSA, = IgG, and = JYM, (c) is the amide I spectral region comparing = egg albumin and = JYM, and (d) is the lipid spectral region (≈1740 cm^{-1}) for = β-lactoglobulin, = α-lactalbumin, = BSA, = IgG, = casein, = egg albumin, and = JYM.

Figure S8 shows the mid-infrared spectrum; showing the lipid (1740 cm^{-1}) and carbohydrate (1080 cm^{-1}) regions, for NitroTech protein powder, BSA, and spikes. From Figure S8 a, it can be seen that as the protein content of BSA is increased, the absorbance maximum of the lipid peak of the protein powder NitroTech at 1743 cm^{-1} decreases. From Figure S8 b, it can be seen that there is little change in the carbohydrate region, going from that of the protein powder NitroTech to the protein standard BSA.

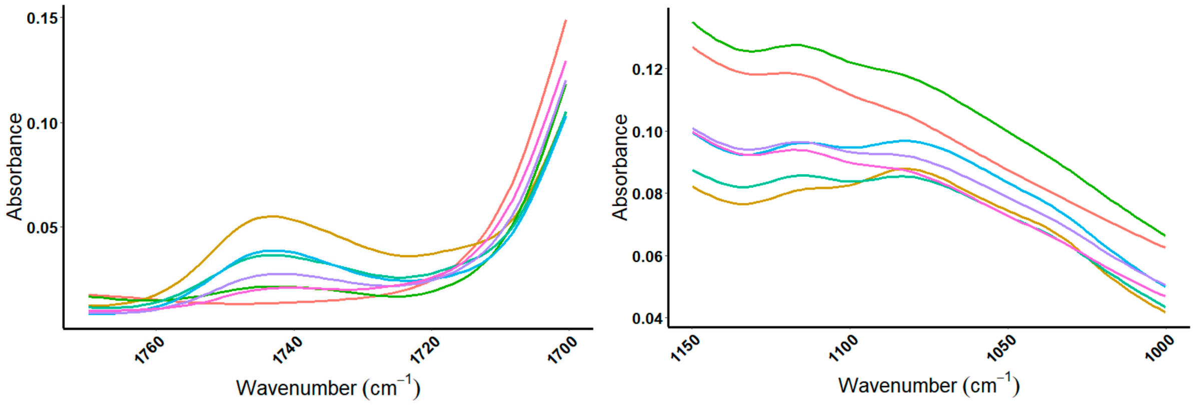

(a) (b)

Figure S8. Lipid and carbohydrate regions of NitroTech protein powder, BSA, and spikes. MIR spectra of NitroTech spiked with increasing levels of BSA from 1:1 to 1:10 in the (a) lipid spectral region (≈1743 cm^{-1}), and (b) carbohydrate spectral region (≈1080 cm^{-1}), where — = NitroTech protein powder, — = NitroTech:BSA(1:2), — = NitroTech:BSA(1:4), — = NitroTech:BSA(1:6), — = NitroTech:BSA(1:8), — = NitroTech:BSA(1:10), — = BSA.
